# Predictors of Response to Fremanezumab in Migraine Patients with at Least Three Previous Preventive Failures: Post Hoc Analysis of a Prospective, Multicenter, Real-World Greek Registry

**DOI:** 10.3390/jcm12093218

**Published:** 2023-04-29

**Authors:** Andreas A. Argyriou, Emmanouil V. Dermitzakis, Georgia Xiromerisiou, Dimitrios Rallis, Panagiotis Soldatos, Pantelis Litsardopoulos, Michail Vikelis

**Affiliations:** 1 Headache Outpatient Clinic, Neurology Department, Agios Andreas State General Hospital of Patras, 26335 Patras, Greece; pantelis84@hotmail.com; 2 Euromedica General Clinic, 54645 Thessaloniki, Greece; manolis.dermitzakis@gmail.com; 3 Department of Neurology, School of Medicine, University of Thessaly, 41221 Larissa, Greece; georgiaxiromerisiou@gmail.com; 4 Department of Neurology, Tzaneio General Hospital of Piraeus, 18536 Athens, Greece; jimrallis@hotmail.com; 5 Private Practice, 24100 Kalamata, Greece; soldatosp@gmail.com; 6 Headache Clinic, Mediterraneo Hospital, 16673 Glyfada, Greece; mvikelis@headaches.gr

**Keywords:** CGRP, monoclonal antibodies, fremanezumab, phenotypes, predictors, response, episodic migraine, chronic migraine

## Abstract

Objective: To define, in a real-world population of patients with high-frequency episodic (HFEM) or chronic migraine (CM), the predictive role of socio-demographic or phenotypic profiling of responders to fremanezumab. Patients and methods: Two-hundred and four adult fremanezumab-treated patients with either HFEM or CM, who failed to at least three preventive treatments, provided data at baseline on several individual socio-demographic and phenotypic variables. These variables were analyzed for their ability to independently predict the response (50–74% response rates) or super-response (≥ 75% response rates) to fremanezumab. Patients were followed from 3–18 months of fremanezumab exposure. Results: The main finding to emerge from univariate analyses was that three baseline socio-demographic/clinical variables, i.e., age group 41–70 years (*p* = 0.02); female gender (*p* = 0.03); patients with HFEM (*p* = 0.001), and three clinical phenotypic variables, i.e., strict unilateral pain (*p* = 0.05); pain in the ophthalmic trigeminal branch (*p* = 0.04); and the “imploding” quality of pain (*p* = 0.05), were significantly related to fremanezumab response. However, in multivariate analysis, only HFEM (*p* = 0.02), the presence of strict unilateral (*p* = 0.03), and pain location in the ophthalmic trigeminal branch (*p* = 0.036) were independently associated with good fremanezumab response. Allodynia (*p* = 0.04) was the only clinical predictive variable of super-responsiveness to fremanezumab. Conclusions: A precise phenotypic profiling with identification of pain characteristics consistent with peripheral and/or central sensitization might reliably predict the responsiveness to fremanezumab in migraine prophylaxis.

## 1. Introduction

The introduction of monoclonal antibodies (MAbs), specifically targeting the calcitonin gene-related peptide (CGRP) or its receptor (anti-CGRP MAbs), has revolutionized the prophylactic treatment of migraine [1]. Their mode of action is based on the ability to selectively inhibit the activation of the trigeminovascular pain pathway [2,3].

Fremanezumab, a humanized anti-CGRP Mab with abilities to selectively target the CGRP ligand and to prevent its binding to the receptor in the trigeminal ganglion and meningeal nociceptors [4], has demonstrated a favorable benefit-risk ratio in large regulatory placebo-controlled randomized clinical trials [5] and was approved in 2018–2019 by international drug agencies for the prophylactic treatment of both episodic (EM) and chronic migraine (CM). After the release of formal approval, several real-world studies worldwide validated its excellent safety/tolerability profile [6,7], marking the onset of a new era in migraine prophylaxis, compared to the usual standard of care with the use of orally taken beta blockers, antiepileptics and tricyclics [8].

Fremanezumab has been commercially available in Greece for migraine prophylaxis since 2020, while reimbursement came in 2021 for patients with high frequency EM (HFEM: 8–14 days/month) or CM, having previously failed to at least three preventives, including OnabotulinumtoxinA (only in CM patients). We have recently reported the outcome of the first prospective real-world study from Greece on the efficacy/safety of fremanezumab in difficult-to-treat migraine patients, and demonstrated that it was able to reduce, by at least 50%, the monthly headache days (MHD) in about two-third of the 204 enrolled patients with either HFEM or CM. As a result of this beneficial effect, patients had less disability and improved quality of life [9].

Our results are generally in agreement with previous evidence showing that up to one-third of patients remain unresponsive to preventative therapies with anti-CGRP MAbs, including fremanezumab [10]. Towards the latter evidence, and also considering the lack of a reliable disease biomarker, it is important to identify clinical predictors of response to anti-CGRP MAbs in order to guide tailored and personalized therapeutic protocols for each patient so as to optimize good clinical outcomes as well as resources allocation [11].

Although there is evidence to suggest that some baseline demographic and clinical characteristics, as well as phenotypic features, might be able to predict the responsiveness to anti-CGRP MAbs, the issue still remains only partly elucidated because of mixed results and common heterogeneities in findings reported from available studies [12,13,14,15]. Another important aspect that needs to be further addressed is to define the profile of patients who experience super-response to anti-CGRP MAbs, especially if potential predictors to these outcomes are to be identified for these individuals.

Therefore, the aim of this post hoc analysis of data extracted from a prospective, multicenter, Greek registry is to define, in a real-world population of patients with HFEM or CM, the predictive role of socio-demographic and phenotypic profiling of responders (≥ 50% MHD reduction) or super-responders (≥ 75% MHD reduction) to fremanezumab.

## 2. Materials and Methods

Two-hundred and four adult patients with a definite diagnosis of either HFEM or CM [16], who received treatment with at least 3 monthly cycles or 1 per trimester cycle of fremanezumab at six different Greek hospitals or headache-focused private clinics, took part in this post hoc analysis. The study protocol was approved by the Institutional Review Board of “Agios Andreas” Patras General Hospital, and an informed consent was obtained from each patient before being included into the study, in accordance with the requirements of the Declaration of Helsinki.

Eligibility was confirmed by a protocol-specific checklist, while the inclusion and exclusion criteria have been previously described in detail [9]. Briefly, patients had to suffer from either HFEM or CM with or without aura or medication overuse headache (MOH) and be scheduled to receive prophylactic treatment with fremanezumab, as per the approved indication/contraindication [17] and current standard Greek clinical practice and national reimbursement policies. Anti-CGRP MAbs naïve patients received subcutaneous fremanezumab (Ajovy^®^ 225 mg/pf-syr, Teva Pharma-Hellas) 225 mg monthly (every 28–30 days) or 675 mg quarterly (every 90 days) for at least 3 months (12 weeks) before establishing the response rates. Hence, patients were followed from 3–18 months of fremanezumab exposure.

The following socio-demographic, clinical variables, and phenotypic characteristics were carefully collected at baseline and were then analyzed for their ability to predict the response to fremanezumab, in line with previous relevant publications [14,18]: gender; age groups in years (41–70 vs. 18–40); migraine type (HFEM vs. CM); BMI status (normal [<24.9] vs. overweight/obese [>25]); number of failed preventives (3–5 vs. 6–7); duration of migraine diagnosis (2–15 vs. above 15 years), presence (yes/no) of MOH; aura; family history and comorbidities, the latter either psychiatric or gastrointestinal. Additionally, patients were asked to report the presence of strict unilateral pain (pain never felt on the other side of the head) vs. alternating side; allodynia, i.e., pain resulting from application of a non-noxious stimulus (yes/no); pain in ophthalmic trigeminal branch (yes/no); prodromal dopaminergic symptoms, i.e., mood changes, yawning, somnolence, drowsiness, food craving (yes/no); unilateral autonomic symptoms, i.e., eye redness, lacrimation, nasal congestion, rhinorrhea, eyelid edema, facial edema, forehead and facial sweating, miosis, ptosis (yes/no); quality of pain (imploding vs. exploding pain); response to triptans, defined as headache resolution within 2 h after triptan intake (yes/no); presence of known migraine triggers, including stress, irregular sleep schedule, specific food/alcohol/caffeine consumption, weather changes, dehydration, and luminous and olfactory stimuli (yes/no); and pericranial muscle tenderness (yes/no).

After the first fremanezumab administration, patients completed a daily headache diary (compliance was set to at least 80% of total monthly days) in paper format, and based on the corresponding recordings, compared to those obtained pre-treatment, we divided them in three groups: non-responders (<50% reduction in MHD); responders (50–74% reduction in MHD) and super-responders (≥75% reduction in MHD). Migraine patients were defined as responders or super-responders if they experience either a >50% or a >75% decrease, respectively, in MHD or in the monthly number of moderate/severe headache days during the last 4 weeks of treatment, compared to baseline. Patients who had a decrease in MHD ranging from 26 to 49%, compared to baseline, are defined as non-responders, while a full non-responder is a patient who experiences a <25% decrease in MHD [19]. For the purpose of our study the latter two groups (non-responders and full non-responders) were merged into one group as “non-responders”.

We then compared the above-mentioned baseline socio-demographic and clinical characteristics, as well as phenotypic profiling, between non-responders vs. responders and responders vs. super-responders in order to define the predictors of response at ≥50% and at ≥75% to fremanezumab.

### Statistical Analysis

To identify predictors of response to fremanezumab, we performed a univariate analysis using baseline demographic and clinical characteristics of their migraine. Patients who responded to fremanezumab, defined as an at least 50% reduction in their MHD, and non-responders (<50% MHD reduction) were compared using the two-sided chi square test with Yate’s correction. The same statistical test was performed to compare patients with response (50–74% MHD reduction) vs. super-response (≥75% MHD reduction) to fremanezumab. To assess independency, all significant variables in univariate analysis were then entered into a backward multivariate logistic regression analysis. All tests were two-tailed and statistical significance was set at the *p* < 0.05 level. Statistical analysis was performed using SPSS for Windows (release 27.0; SPSS Inc., Chicago, IL, USA).

## 3. Results

The flow chart, as well as the demographic and baseline clinical migraine characteristics of our study sample included in this post hoc analysis, are described in detail in our primary publication that contained our results on the efficacy/safety of fremanezumab in migraine prophylaxis [9]. Briefly, there were 210 patients initially enrolled, with the majority of them to be able to complete the study. There were 6 cases of early withdrawal from the study for reasons including, lost to follow-up (n= 3); cases remained in significant remission and individually decided not to continue treatment (n=2), as well as one case of pregnancy. As such, of a total of 204 fremanezumab-treated patients for either HFEM (n = 97; 47.5%) or CM (n = 107; 52.4%), after having previously failed a median of 5 preventives, 171 (83.8%) were females, and they had a median age of 47.5 years. The majority (n = 131; 64.3%) of them had a normal BMI of <24.9 and were diagnosed with concurrent MOH (n = 122; 59.8%). Psychiatric comorbidities were also common (n = 121; 59.3%). A total of 148 patients (81/97; 83.5% with HFEM and 67/107; 62.6% CM patients) obtained an at least 50% reduction in MHD, compared to baseline, and were counted as treatment responders.

### 3.1. Comparison of Baseline Demographic and Clinical Characteristics as Well as Phenotypic Profiling between Responders vs. Non-Responders to Fremanezumab

Concerning the comparison in baseline demographics and clinical features, the responders were more frequently females (*p* = 0.03), aged between 41–70 years (*p* = 0.02), who received fremanezumab for HFEM (*p* = 0.001) than non-responders. The rest of the baseline demographic and clinical data were well balanced between the two groups, as none of the analyzed variables were found to have a statistically significant association with occurrence of response vs. non-response to fremanezumab, including the family history of migraine; BMI status; the number of previously failed preventives; the duration of migraine diagnosis; and the occurrence of MOH, aura, or other major comorbidities (Table 1).

After univariate analysis, three variables extracted from the phenotypic clinical profile of patients were related to higher rates of response to fremanezumab and thus to favorable outcomes. The responders presented more frequent strict unilateral pain (odds ratio [OR]: 1.8; 95% confidence interval [CI]: 1.2–3.9; *p* = 0.05) or pain in the ophthalmic trigeminal branch (OR: 3.6; 95% confidence interval [CI]: 1.9–7.1; *p* = 0.04), while the quality of their pain was more frequently described as being “imploding” (OR: 1.7; 95% CI: 0.9–3.1; *p* = 0.05), compared to non-responders (Table 2).

Notably, the presence of allodynia showed a marked trend to significance towards association with a clinically meaningful response to fremanezumab (OR: 1.5; 95% CI: 0.6–3.3; *p* = 0.071).

We finally turned to multivariate analysis to identify the independent predictors of adequate response to fremanezumab (only significant variables were included), and we confirmed this independent association only for HFEM (OR of 3.3; 95% CI: 2.3–5.3; *p* = 0.02) coupled with the presence of strict unilateral pain (OR of 2.1; 95% CI: 1.5–4.3; *p* = 0.03) or pain in the ophthalmic trigeminal branch (OR of 2.6; 95% CI: 1.3–7.3; *p* = 0.036).

### 3.2. Phenotypic Characteristics Comparison between Responders vs. Super-Responders to Fremanezumab

Among a total of 148 responders obtaining an at least 50% reduction in MHD after fremanezumab therapy, 83 responded at 50–74% and 65 at ≥75%, compared to baseline, and were as such classified as either responders or super-responders, respectively. Super-responders more frequently presented allodynia both in univariate (OR of 2.4; 95% CI: 1.2–4.8; *p* = 0.022) and multivariate logistic regression (OR of 2.1; 95% CI: 1.4–6.8; *p* = 0.04) analyses, compared to responders, while all other associations failed to reach significance (Table 3).

## 4. Discussion

The current post hoc analysis sought to prospectively assess the value of several baseline socio-demographic/clinical parameters or phenotypic profiling in predicting the responders (50–74% response rates) or super-responders (≥ 75% response rates) to fremanezumab. The main finding to emerge from univariate analyses was that three from the baseline socio-demographic and clinical variables, i.e., age group 41–70 years (*p* = 0.02); female gender (*p* = 0.03); patients with HFEM (*p* = 0.001), and three variables extracted from the phenotypic clinical profile of patients, i.e., strict unilateral pain (*p* = 0.05); pain in the ophthalmic trigeminal branch (*p* = 0.04); and the “imploding” quality of pain (*p* = 0.05), were significantly related to fremanezumab response. However, in multivariate analysis, only HFEM (*p* = 0.02); the presence of strict unilateral rather than alternating pain (*p* = 0.03); and pain location in the ophthalmic trigeminal branch (*p* = 0.036) were independently associated with good response to fremanezumab. Moreover, allodynia (*p* = 0.04) was the only clinical phenotypic variable that was able to positively and independently predict super-responsiveness to fremanezumab.

Our findings, overall, bolster the argument that symptoms related to both peripheral sensitization, i.e., strict unilateral pain and pain location in the ophthalmic trigeminal branch, and also central sensitization, i.e., allodynia, may be associated with good clinical response to fremanezumab. As such, we can assume that its preventive effects are conveyed via the modulation of overactive somatosensory processing and pain thresholds through activation of the trigeminoautonomic reflex [20], while patients with certain migraine phenotypes, characterized by location of pain strictly unilaterally or specifically in the V1 dermatome, may mostly benefit even during the phase of migraine chronification [21]. In addition, it seems that fremanezumab is able to inhibit the sensitization of centrally situated second-order nociceptive neurons [20], and as such patients with allodynia, a feature consistent with central sensitization, may indeed super-respond to fremanezumab [22,23].

Our results are in agreement with previous publications demonstrating that the responsiveness to anti-CGRPs was positively associated with symptoms related to both peripheral and central sensitization [12,13,14]. The relevance of migraine type, i.e., HFEM over CM, in predicting the therapeutic response to anti-CGRP MAbs has been pointed out also by other research groups, demonstrating that fewer migraine days at baseline was associated with good response [24]. Our findings also partly support the hypothesis that the subjective perception of pain as an “imploding” headache, compared to “exploding” pain, might be a feature with some ability to predict the response to anti-CGRPS; consistent with a similar good response which was previously seen with onabotulinumtoxin-A [25].

However, we were unable to confirm findings from other studies, which favor the role of several other variables in predicting the responsiveness to anti-CGRPs, such as dopaminergic symptoms; autonomic symptoms; absence of psychiatric comorbidities; good response to triptans; normal BMI; age at migraine onset; family history of migraine; number of failed preventive medications; and MIDAS score [12,13,15,24,26,27,28]. Methodological differences, including populations investigated, sample sizes, and clinical efficacy outcomes studied, may account for discrepancies between results of available studies.

Nonetheless, it should be emphasized that our study comprised a homogenous sample of fremanezumab-treated patients with difficult-to-treat migraine, having at least three previously failed preventive treatments. The latter, in our opinion, should be counted among the strengths of our study, as other relevant publications attempted to identify predictors of response after exposure to mixed antiCGRP MAbs, including erenumab, fremanezumab, and galcanezumab, according to drug market availability or physician’s choice [14,29]. In any case, we cannot exclude the presence of other significant predictive socio-demographic or clinical variables than those included in our analysis, and as such a more in-depth clinical profiling may discover other strong predictors of response to fremanezumab.

To explain why we only provide data about predictors of response on no anti-CGRP MAbs for migraine prophylaxis other than fremanezumab, we should mention that fremanezumab was approved first for reimbursement, according to the national policies concerning reimbursement of expensive therapies for migraine (early access release date in late 2020 and formal approval in July 2021) in Greece. Erenumab and galcanezumab received a similar approval quite recently, in February 2022 and in February 2023, respectively. Eptinezumab is currently unavailable in Greece. Both fremanezumab, erenumab and galcanezumab are currently fully reimbursed by the National Health System and social services in Greek patients with HFEM or CM who failed at least three preventive treatments, including OnabotulinumtoxinA in patients clinically classified as having CM [30]. Patients with private insurance that covers the cost of anti-CGRP MAbs also have access to these treatments.

According to international but also national guidelines on the use, monitoring and discontinuation of anti-CGRP MAbs, it is recommended to treat adult patients with 4 or more migraine days per month for at least 3 months before establishing efficacy. With a reduction of >50% in monthly headache days compared to baseline, it is advised to further continue treatment for up to 12–18 months of therapy [19,30,31], and then pause for 1–2 months to monitor for a migraine relapse; in such cases, re-administration of the discontinued anti-CGRP MAb is recommended [19,30,31]. However, in case of 30% of monthly headache days, compared to baseline, after 3 months of therapy, it is advised to continue exposure for another 3 months before concluding on the efficacy of a given anti-CGRP MAb [19,30] Nonetheless, if a reduction of <30% in monthly headache days occurs after 6 months of continuous treatment with the first-line anti-CGRP MAb, it is recommended to switch to another anti-CGRP MAb with different target upon CGRP, i.e., CGRP ligand or CGRP receptor) [32] or to commence dual targeting with onabotulinumtoxinA add-on to anti-CGRP MAb in these treatment-refractory patients [33,34], as a delayed clinically meaningful response is unlikely to occur with further (after 6 months) exposure to initial treatment with the use of either monoclonal antibodies targeting the CGRP ligand or its receptor [35]. A quite recently published report contradicts the latter view, by demonstrating that late responses to anti-CGRP MAbs may occur even beyond 12 months of continuous treatment [36]. Further studies on this clinically important issue are warranted before definite conclusions can be drawn. 

## 5. Conclusions

To conclude from a clinical point of view, our results indicate that a precise phenotypic profiling with identification of pain characteristics consistent with peripheral and/or central sensitization might be able to predict responsiveness to fremanezumab in migraine prophylaxis. Further larger prospective studies, including genetic sequencing and biomarker profiling, are warranted to address the important issue concerning a precise prediction of response to available anti-CGRPs MAbs.

## Figures and Tables

**Table 1:** Demographic and baseline disease’s clinical data in responders (at least 50% reduction in MHD) vs. non-responders (<50% MHD reduction) to fremanezumab. *p* values in bold indicates statistical significance.

	Responders		Non-Responders			
	n = 148		n = 56			
Predictors	N	%	N	%	O.R (95% CI)	*p* Value
Age in years 41–70 vs. 18–40	95	64.2	21	37.5	1.2 (0.8–1.6)	**0.02**
Gender Females vs. Males	131	88.5	40	71.4	3.2 (1.1–9.5)	**0.03**
Migraine type HFEM vs. CM	81	54.7	16	28.6	7.3 (3.1–8.6)	**0.001**
BMI status						
Normal (<24.9) vs. Overweight/obese (>25)	98	66.2	33	58.9	0.7 (0.4–1.4)	0.332
Failed preventives (n) 3–5 vs. 6–7	65	43.9	20	35.7	0.8 (0.3–1.5)	0.473
Duration in migraine diagnosis (years) 2–15 vs. above 15	74	50	21	37.5	0.9 (0.7–1.2)	0.374
MOH Yes vs. No	90	60.8	32	57.1	0.6 (0.2–1.9)	0.432
Aura Yes vs. No	19	12.8	10	17.8	0.6 (0.3–1.2)	0.3
Family History Yes vs. No	63	42.5	31	53.5	0.8 (0.7–1.1)	0.12
Comorbidities Yes vs. No						
Psychiatric	85	57.4	38	67.8	0.6 (0.3–1.2)	0.151
Gastrointestinal	28	18.9	13	23.2	0.9 (0.8–1.1)	0.513

**Table 2:** Incidence of various clinical predictors in migraine patients with response (at least 50% reduction in MHD) vs. non-response (<50% MHD reduction) to fremanezumab.

	Responders		Non-Responders			
Predictors	n = 148		n = 56		O.R (95% CI)	*p* Value
	N	%	N	%		
Strict unilateral pain	60	40.5	15	26.8	1.8 (1.2–3.9)	**0.05**
Allodynia	52	35.1	13	23.2	1.5 (0.6–3.3)	0.071
Pain in ophthalmic trigeminal branch	30	20.3	5	8.9	3.6 (1.9–7.1)	**0.04**
Prodromal Dopaminergic symptoms	85	57.4	25	44.6	0.6 (0.4–1.2)	0.361
Unilateral Autonomic symptoms	56	37.8	15	26.8	0.8 (0.7–1.5)	0.117
Imploding vs. exploding pain	85	57.4	20	35.7	1.7 (0.9–3.1)	**0.05**
Response to triptans Yes vs. No	102	68.9	36	64.2	0.9 (0.4–1.9)	0.513
Presence of triggers Yes vs. No	71	47.9	24	42.9	0.7 (0.4–1.2)	0.706
Pericranial Muscle tenderness Yes vs. No	79	53.4	26	46.4	0.5 (0.5–1.7)	0.463

*p* values in bold indicates statistical significance.

**Table 3:** Incidence of various clinical predictors in migraine patients with response (50–74% reduction in MHD) vs. super-responders (≥75% MHD reduction) to fremanezumab.

	Responders		Super-Responders			
Predictors	n = 81		n = 67		O.R (95% CI)	*p* Value
	N	%	N	%		
Strict unilateral pain	32	39.5	28	41.8	0.7 (0.4–1.4)	0.544
Allodynia	21	25.9	31	46.2	2.4 (1.2–4.8)	**0.022**
Pain in ophthalmic trigeminal branch	18	22.2	12	17.9	0.8 (0.3–1.7)	0.681
Dopaminergic symptoms	48	59.2	37	55.2	0.5 (0.3–1.4)	0.323
Unilateral Autonomic symptoms	33	40.7	23	34.3	0.6 (0.3–1.5)	0.364
Imploding vs. exploding pain	47	58.1	38	56.7	0.8 (0.4–1.7)	0.733
Response to triptans Yes vs. No	59	72.8	43	64.2	1.0 (0.5–2.5)	0.203
Presence of triggers Yes vs. No	38	46.9	33	49.2	0.8 (0.3–1.4)	0.871
Pericranial Muscle tenderness Yes vs. No	44	54.3	35	52.2	0.8 (0.5–1.7)	0.743

*p* values in bold indicates statistical significance.

## Data Availability

The data that support the findings of this study are available from the corresponding author upon reasonable request.
